# Small Molecule Injection into Single-Cell *C. elegans* Embryos via Carbon-Reinforced Nanopipettes

**DOI:** 10.1371/journal.pone.0075712

**Published:** 2013-09-26

**Authors:** Lucy D. Brennan, Thibault Roland, Diane G. Morton, Shanna M. Fellman, SueYeon Chung, Mohammad Soltani, Joshua W. Kevek, Paul M. McEuen, Kenneth J. Kemphues, Michelle D. Wang

**Affiliations:** 1 Department of Physics - Laboratory of Atomic and Solid State Physics, Cornell University, Ithaca, New York, United States of America; 2 Howard Hughes Medical Institute, Cornell University, Ithaca, New York, United States of America; 3 Department of Molecular Biology and Genetics, Cornell University, Ithaca, New York, United States of America; University of North Carolina at Chapel Hill, United States of America

## Abstract

The introduction of chemical inhibitors into living cells at specific times in development is a useful method for investigating the roles of specific proteins or cytoskeletal components in developmental processes. Some embryos, such as those of *Caenorhabditis elegans*, however, possess a tough eggshell that makes introducing drugs and other molecules into embryonic cells challenging. We have developed a procedure using carbon-reinforced nanopipettes (CRNPs) to deliver molecules into *C. elegans* embryos with high temporal control. The use of CRNPs allows for cellular manipulation to occur just subsequent to meiosis II with minimal damage to the embryo. We have used our technique to replicate classical experiments using latrunculin A to inhibit microfilaments and assess its effects on early polarity establishment. Our injections of latrunculin A confirm the necessity of microfilaments in establishing anterior-posterior polarity at this early stage, even when microtubules remain intact. Further, we find that latrunculin A treatment does not prevent association of PAR-2 or PAR-6 with the cell cortex. Our experiments demonstrate the application of carbon-reinforced nanopipettes to the study of one temporally-confined developmental event. The use of CRNPs to introduce molecules into the embryo should be applicable to investigations at later developmental stages as well as other cells with tough outer coverings.

## Introduction

The ability to introduce chemical inhibitors into single cells has been an important approach for understanding signaling pathways in many organisms. However, some cells, such as those of the *Caenorhabditis elegans* embryo, are surrounded by an outer layer that provides protection from the environment and makes application of drugs challenging. To address this difficulty, we have developed an injection technique using carbon-reinforced nanopipettes (CRNPs) to introduce a chemical inhibitor into the single-celled *C. elegans* embryo and have used this approach to reinvestigate early polarity establishment. Our technique allows penetration of the embryo with minimal cellular damage, at precisely controlled times in development, facilitating the study of temporally-confined cellular events.

The single-celled *C. elegans* embryo is well established as a model system for studying cell polarity. During the first cell cycle, a remarkable reorganization of the cytoskeletal and cytoplasmic components occurs, culminating in an asymmetric first division yielding daughter cells with different sizes, cell cycle rates and developmental potential [1]. In the early one-cell embryo the essential polarity proteins PAR-3, PAR-6, and PKC-3 are present uniformly around the cortex, but concomitant with actomyosin-driven cortical flows, recede from the posterior end and occupy a cortical domain in the anterior half of the embryo [2,3,4,5,6]. The regression of the anterior PAR proteins away from the posterior pole allows a second set of essential polarity proteins, PAR-2 and PAR-1, to localize to the posterior cortex, in a manner that is mutually exclusive with the anterior proteins [7,8,9], reviewed by Cowan & Hyman [10], Nance & Zallen [11], and Noatynska & Gotta [12]. It is clear that actomyosin contractility plays a significant role in anteroposterior (A-P) polarization [2,10,13] and there is evidence that microtubules can also direct polarity initiation in the early embryo [14,15,16,17].

A number of experimental approaches have been used to address the role of the cytoskeleton in *C. elegans* embryo polarity, including RNAi knockdown of individual proteins, genetic analyses, and treatment with chemical inhibitors and each system has inherent limitations. Because many cytoskeletal components are essential, using RNAi knockdown and genetic mutation to probe processes of polarity can reduce viability and/or result in sterility, yielding few embryos for analysis, and those remaining may have only partial depletion of protein activity. The use of chemical inhibitors to perturb the early embryo has been difficult due to the tough eggshell covering and permeability barrier that surround the *C. elegans* embryo [18,19,20]. Permeablilization of the embryo for exposure to specific drugs [19,21,22,23,24] has been exceptionally challenging in very early embryos due to their fragility. Genetic mutants and RNAi knockdown to produce *C. elegans* embryos with permeable eggshells have simplified embryonic drug treatment [25,26], but it would also be useful to directly introduce inhibitors into embryos of any genotype. Glass micropipettes have been used to pierce the *C. elegans* embryo for introduction of dyes by iontophoresis to study cell-cell communication in *C. elegans* [27,28]. However, glass needles are quite fragile, and this approach has not generally been employed for delivery of drug delivery in *C. elegans*; investigators have instead relied upon the other means of eggshell permeabilization, noted above. We have found that by reinforcing glass pipettes with an interior lining of carbon, their use for injection of molecules into *C. elegans* embryos becomes readily successful and reproducible. The injection technique that we have developed enables direct administration of small molecules at an extremely early developmental stage, and can be used for embryos of any genotype to perturb developmental events with high temporal accuracy and reproducibility.

We have utilized carbon-reinforced nanopipettes (CRNPs) to penetrate the *C. elegans* embryo and directly introduce small molecules, and have coupled this with live imaging to visualize the effects of specific inhibitor treatment. In addition to the precise temporal control of this approach, our injection technique allows for simple dosage titration and can easily be combined with RNAi and genetic mutation. Because of the very fine tip of these pipettes, injection into single blastomeres at later stages is also possible. To demonstrate the utility of the CRNPs we have injected the actin polymerization inhibitor, Latrunculin A (LatA), into one-cell embryos prior to polarity establishment and have determined the consequences of such perturbations on the dynamic localization of two critical polarity proteins, the posterior protein PAR-2 and the anterior protein PAR-6.

## Results

### Carbon-reinforced nanopipettes (CRNPs) as a novel drug delivery tool

Single-cell microinjection has been utilized quite commonly as a method for directly introducing small molecules, proteins, RNAs, and DNAs into individual cells. Micropipettes have a tip size of approximately 0.5 µm [29] and are fabricated by pulling glass capillaries to the desired tip diameter [30]. These micropipettes have several limitations owing to the material properties of glass and their large tip size, relative to cell size. Cellular damage due to the large pipette tip diameter is a major limitation in microinjection techniques [31], especially when the cells are small. Reducing the tip diameter should reduce cellular damage. However, glass pipettes, when pulled to smaller dimensions, are more fragile and prone to breakage [32].

**Figure 1:**
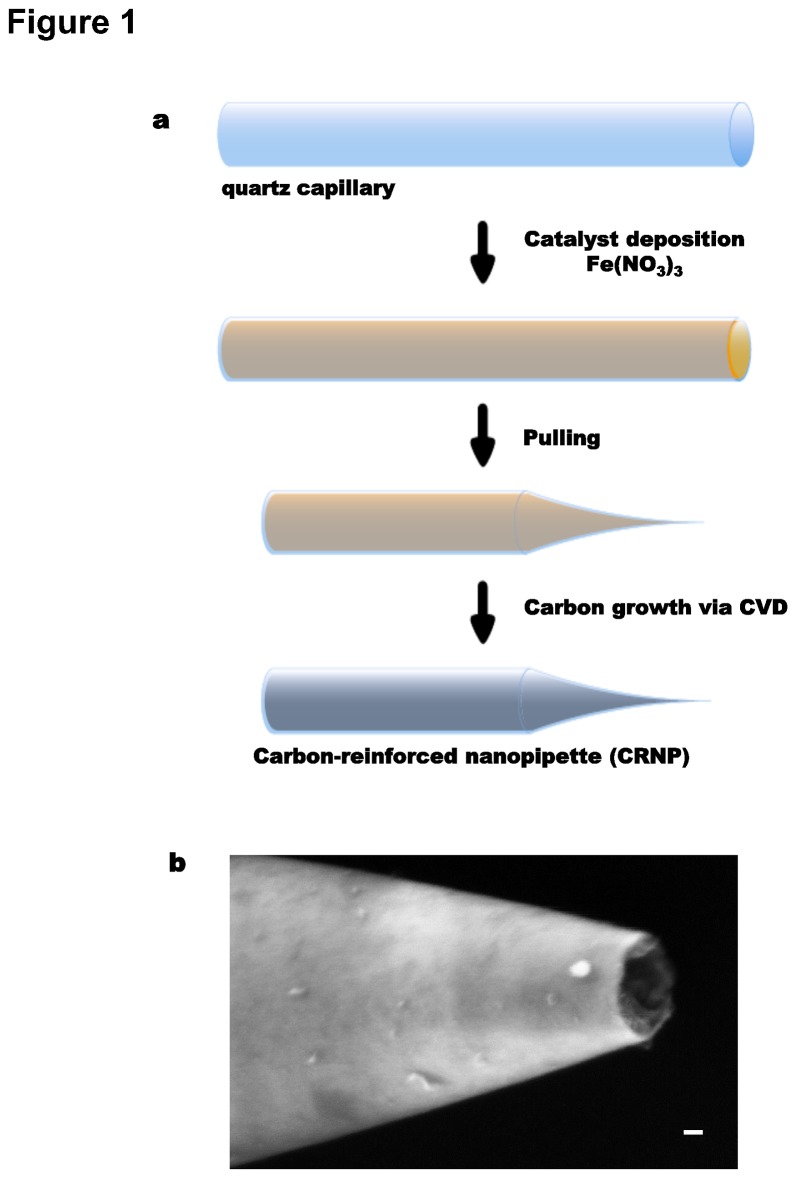
Carbon-reinforced nanopipette (CRNP) fabrication. (**a**) CRNP fabrication procedure. Quartz capillaries were filled with Fe(NO_3_)_3_ catalyst and left to dry, then pulled into pipettes of desired geometry. Carbon was grown within the pipette using chemical vapor deposition (CVD). (**b**) A SEM image of the tip of a CRNP, scale bar is 20 nm.

To successfully pierce the chitinous shell and permeability barriers, while minimizing damage to the embryo, the tip of an injection pipette must be both strong and small. To accomplish this, quartz capillaries were pulled to an outer tip diameter of 135 ± 66 nm (mean ± SD) and then reinforced with an interior layer of carbon (Figure 1a and b). Such pipettes are less prone to breakage after repeated injections than are unlined capillaries. An added advantage of the carbon coating is increased visibility of the tip location of the CRNP. Carbon nanopipettes have previously been used to inject dyes [32] and secondary messengers [33] into epithelial cells. Here we have optimized their geometry for precise and delicate injection into the single-cell C*. elegans* embryo.

**Figure 2:**
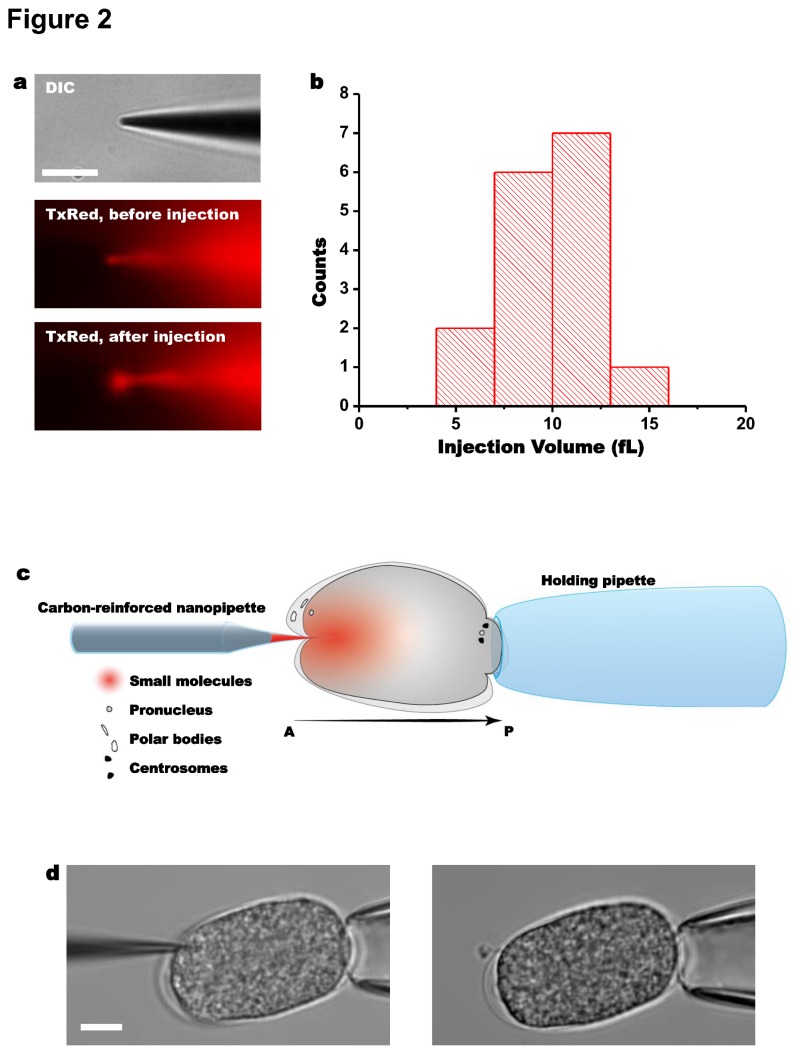
CRNP injection characterization and experimental configuration. (**a**) Characterization of injection volume. A differential interference contrast (DIC) image of a CRNP and fluorescence images before injection of dextran-TxRed into a droplet of glycerol and immediately after the injection. (**b**) Histogram of injection volume from multiple injections using one representative CRNP. (**c**) A cartoon and a (**d**) DIC image of the experimental configuration for embryo injection. A quartz holding pipette was used to immobilize the embryo during injection. Light suction applied through the holding pipette also allowed for the withdrawal of the CRNP. In all figures, unless otherwise stated, *t* = 0 is defined as the beginning of meiosis II, injection occurs at t ^≈^ 0: 15, anterior is to the left, and scale bars are 10 µm.

To determine injection volume of our CRNPs, we injected fluorescent dextran into a glycerol droplet (Materials and Methods) (Figure 2a and b). By measuring the fluorescence intensity immediately after injection, the injection volume was estimated to be ~10 femtoliters (fL), representing ~0.1% of the total embryo volume. Multiple injections from the same CRNP resulted in consistent injection volumes. To facilitate the injection procedure into an embryo, we used larger quartz ‘holding’ pipettes to allow withdrawal of the CRNP from the embryo after drug delivery, as presented in Figure 2c and d. The CRNP can be used for repeated injections over the course of days by purging the contents and storing the pipettes in a humid chamber. A video showing an injection in real time is shown in Video S1.

**Figure 3:**
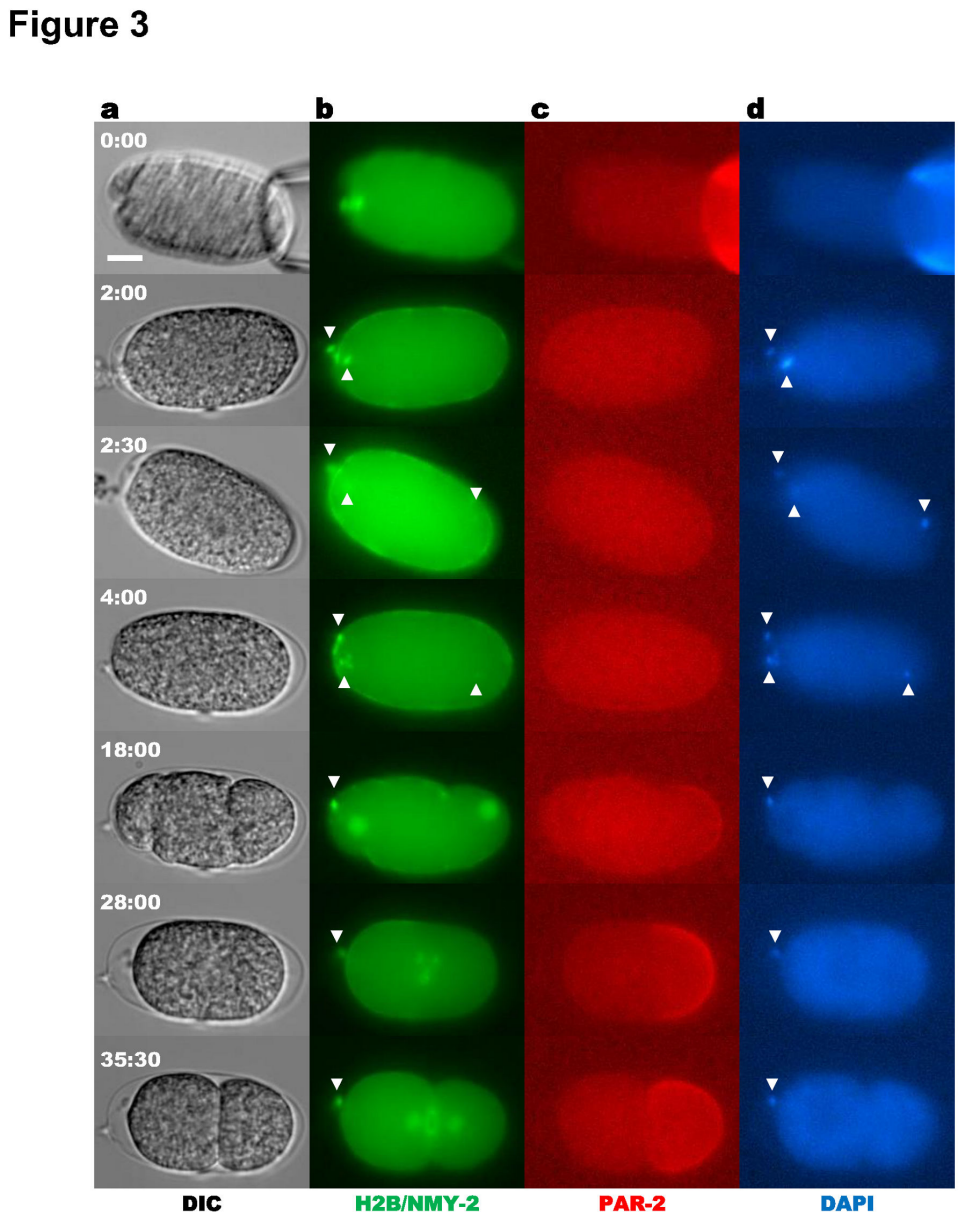
Injection of DAPI into a *C. elegans* embryo. Embryo injected with 0.2 mg/mL DAPI (*N* = 6). (**a**) DIC images. Fluorescence images of (**b**) NMY-2::GFP/ GFP::H2B, (**c**) mCherry::PAR-2, and (**d**) DAPI. White arrowheads indicate co-localization of H2B and DAPI.

As a demonstration of the injection procedure, we injected the DNA intercalating dye, 4’, 6’ diamidino-2-phenylindole dihydrochloride (DAPI), into early one-cell embryos. To ascertain whether these injections perturbed cellular development, the cell cycle, actomyosin contractility, and embryo polarization were monitored in embryos expressing the transgenes GFP::histone 2B (H2B), non-muscle myosin (NMY-2)::GFP, and mCherry::PAR-2 (Figure 3; *N* = 6). Neither the UV excitation of DAPI, nor occasional cytoplasmic leakage that occurred upon CRNP withdrawal had any measurable effects on cell cycle events, polarity establishment, or cytokinesis. DAPI was observed to co-localize with GFP::H2B (Figure 3, white arrowheads) indicating successful injection. Over time the DAPI signal diminished, likely due to photobleaching and dilution of the dye as the pronuclei swelled. Our successful injection of DAPI demonstrated that small molecules could be successfully introduced into the cell via CRNPs without perturbing cellular integrity or development (6 out of 6 embryos).

**Figure 4:**
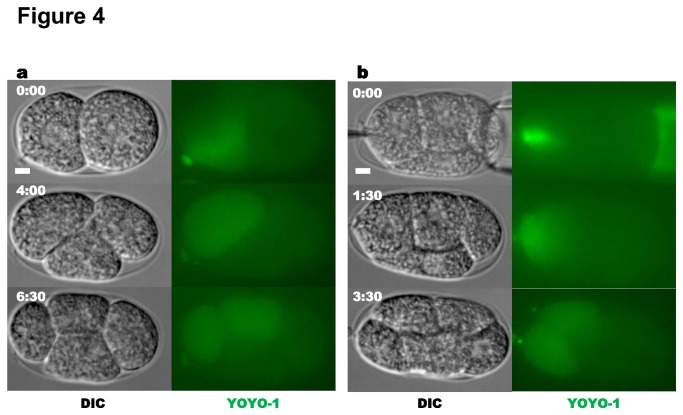
Injection of YOYO-1 into multi-cell embryos. (a) Two-cell embryo in which the P1 blastomere has been injected with 1 µM YOYO-1. (**b**) Four-cell embryo in which the ABa blastomere has been injected with 1 µM YOYO-1.

In order to test whether our technique could be applied to individual cells at later developmental stages, we also injected two-cell and four-cell embryos with the fluorescent dye YOYO-1. YOYO-1 is membrane impermeable [34], and thus should remain confined to the injected blastomere and segregate to its descendants in subsequent cell divisions. YOYO-1 injected via CRNPs into single blastomeres of two-cell or four-cell stage embryos is exclusively localized within the injected cell (Figure 4; N= 5 for 2-cell stage, N= 4 for 4-cell stage). These embryos can complete embryogenesis and hatch into L1 larvae.

### Microfilament Depolymerization Inhibits Embryo Polarization

To further test whether our CRNP injection technique can deliver specific inhibitors in a controlled manner, we chose to inject Latrunculin A into the early embryo, at a time point before polarity is initiated. LatA sequesters actin monomers, reducing the concentration of free actin and effectively inhibiting microfilament polymerization [35]. The temporal correlation between actomyosin clearing in the posterior and PAR-2 localization originally prompted the hypothesis that actomyosin contractility plays a role in embryonic polarity [2,3,8,36]. Classical experiments by Strome and Wood in 1983 first demonstrated that microfilaments were essential for some aspects of polarity, including segregation of germline granules [21]. Additional genetic and biochemical work has also indicated cross-talk between the actomyosin network and PAR proteins [2,37,38,39,40,41,42]. However, other work has suggested the existence of a secondary, microtubule-mediated, polarization pathway [15,43]. We used CRNP mediated drug delivery to re-examine the role of microfilaments in polarity establishment.

**Figure 5:**
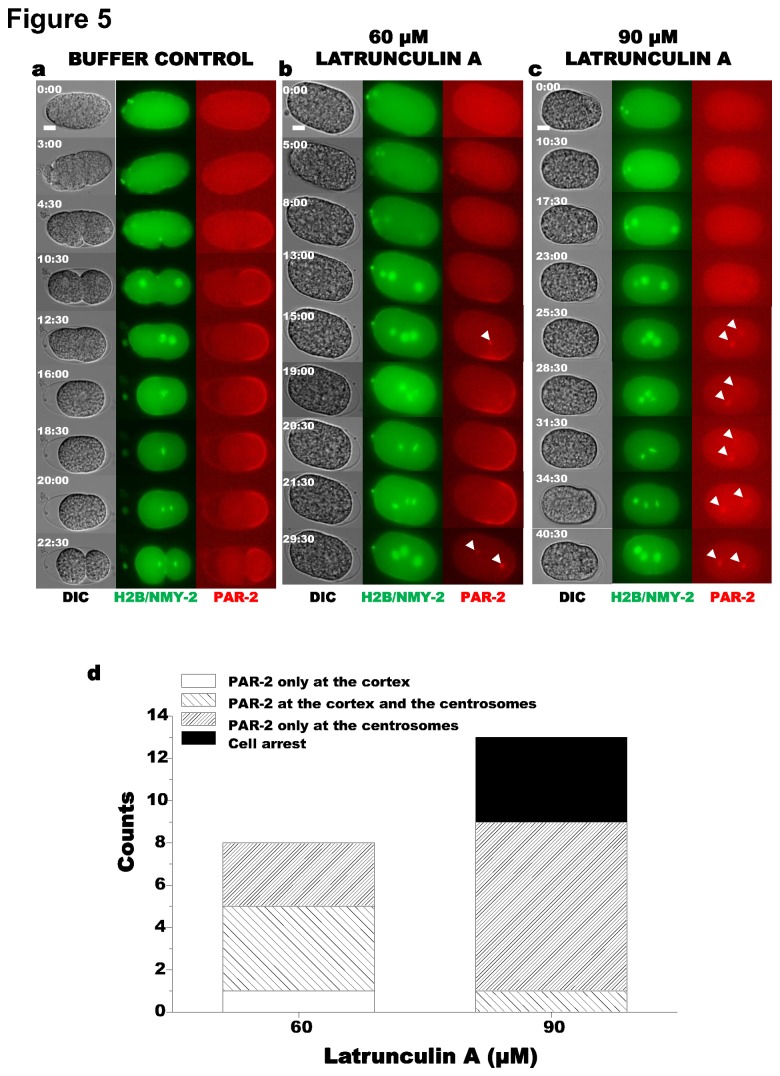
Effect of Latrunculin A concentration on PAR-2 localization. (**a**) Control with injection buffer containing 3.75% DMSO in 0.8x EB (*N* = 10). (**b**) Injection of 60 µM LatA (*N* = 8). Shown is an example embryo in which PAR-2 localized at both the cortex and the centrosomes. (**c**) Injection of 90 µM LatA (*N* = 13). Shown is an example embryo with PAR-2 localization at the centrosomes only. For each panel, columns from left to right: DIC; NMY2::GFP/GFP::H2B, and mCherry::PAR-2. In some images, only one centrosome is in focus. (**d**) Distribution of PAR-2 localization phenotypes after injection of 60 µM or 90 µM LatA. In this and all subsequent figures, rows of images are aligned by nuclear dynamics, as assessed by GFP::H2B.

We injected LatA into single-cell embryos expressing GFP::H2B, NMY-2::GFP, and mCherry::PAR-2, at meiosis II, before polarization of PAR proteins is initiated. Injections of either 60 or 90 µM LatA resulted in loss of cortical contraction, no pseudocleavage, and failed cytokinesis, as well as failure to properly localize PAR-2 (Figure 5b and c). PAR-2 localization showed a clear LatA dosage dependence, with localization patterns falling into three categories: accumulation of PAR-2 exclusively at the cortex (similar phenotype to the control), PAR-2 accumulation at both the cortex and centrosome (moderate phenotype) (Figure 5b), and PAR-2 accumulation exclusively at the centrosome (strong phenotype) (Figure 5c). As delineated in Figure 5d, increasing LatA injection concentration shifted the distribution towards stronger phenotypes. The injection of 60 µM LatA resulted in nearly equal numbers of embryos with moderate and strong phenotypes. Increasing the concentration of LatA to 90 µM resulted in a predominance of embyros with the strong phenotype (Figure 5d). At the injection concentration of 90 µM, four out of thirteen embryos arrested before pronuclear migration, suggesting this dose was near the threshold of LatA toxicity. These embryos were excluded from further analysis. 8 out of 9 of the surviving embryos exhibited PAR-2 accumulation exclusively at the centrosomes, instead of proper posterior cortical localization. Our PAR-2 localization observations are in agreement with those previously reported for LatA [38], although the strong phenotype we observe is more extreme. Our ability to carefully titrate the drug dosage has revealed subtle dynamics of cortical PAR-2. At the 60 µM LatA concentration, PAR-2 was capable of initially localizing to the cortex but over time moved to the centrosomes (Figure 5b).

**Figure 6:**
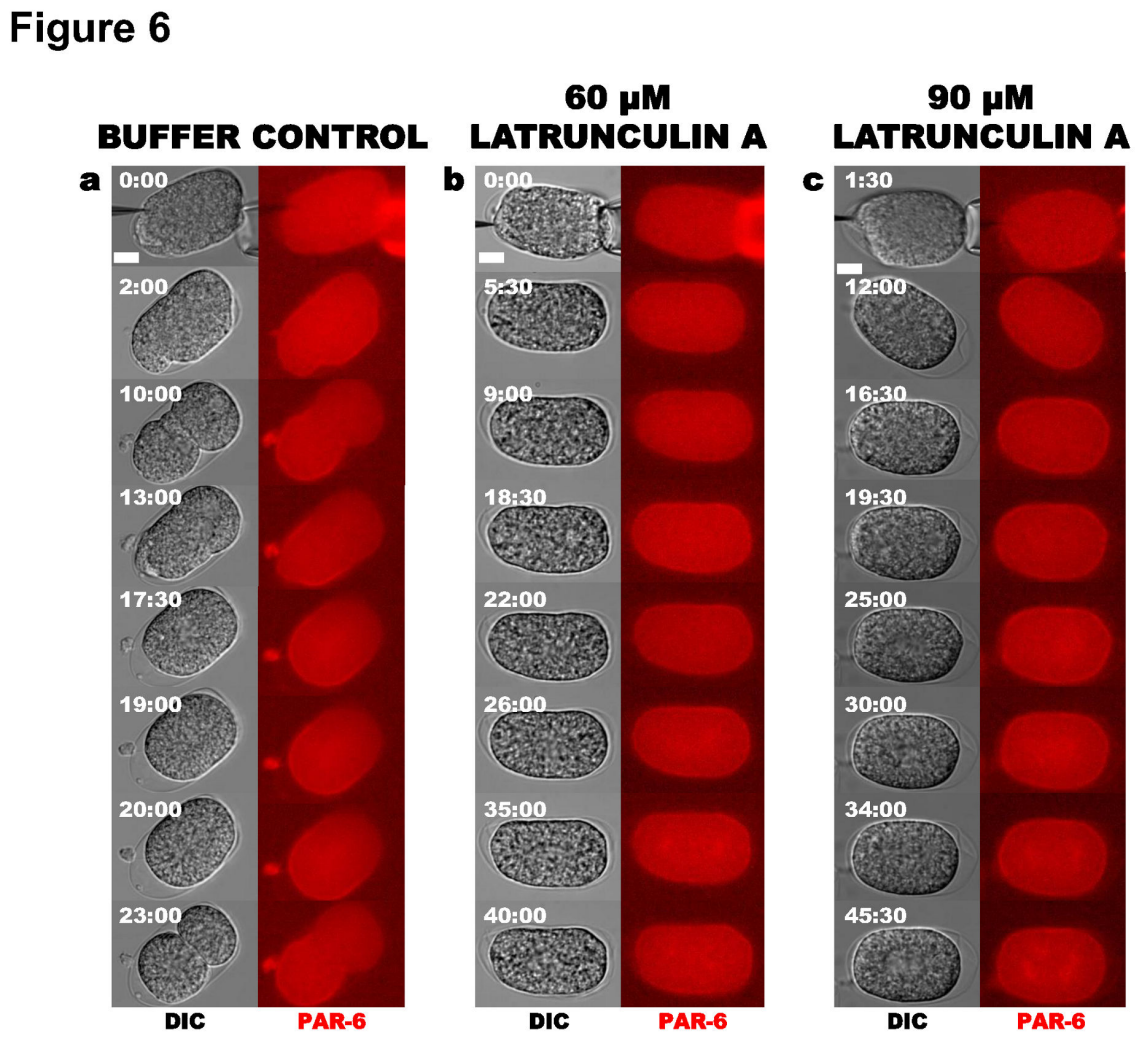
Effect of Latrunculin A on PAR-6 localization. (**a**) Control with injection buffer containing 3.75% DMSO (*N* = 9). (**b**) Injection of 60 µM (*N* = 10) and (**c**) 90 µM (*N* = 5) LatA. In each panel, DIC is on the left and PAR-6::mCherry is on the right.

We also assessed the ability of PAR-6 to localize appropriately following LatA treatment (Figure 6). In control embryos, PAR-6 was initially present around the entire cortex and then receded to the anterior end, concurrent with cortical flows and posterior cortical smoothing (9 out of 9 embryos; Figure 6a). After injection of LatA, no polarization was observed: PAR-6 remained throughout the cortex (Figure 6b and c) correlating with the absence of PAR-2 at the posterior (Figure 5c). Thus LatA treatment does not perturb any process required for PAR-6 cortical accumulation. In 5 out of 8 embryos injected with 60 µM LatA and 5 out of 5 embryos injected with 90 µM LatA, PAR-6 also showed weak accumulation at the centrosomes. Our observations support the conclusion that a functional actin network is necessary for the active segregation of PAR-6 to the anterior cortex, allowing the subsequent loading of PAR-2 onto the posterior cortex.

**Figure 7:**
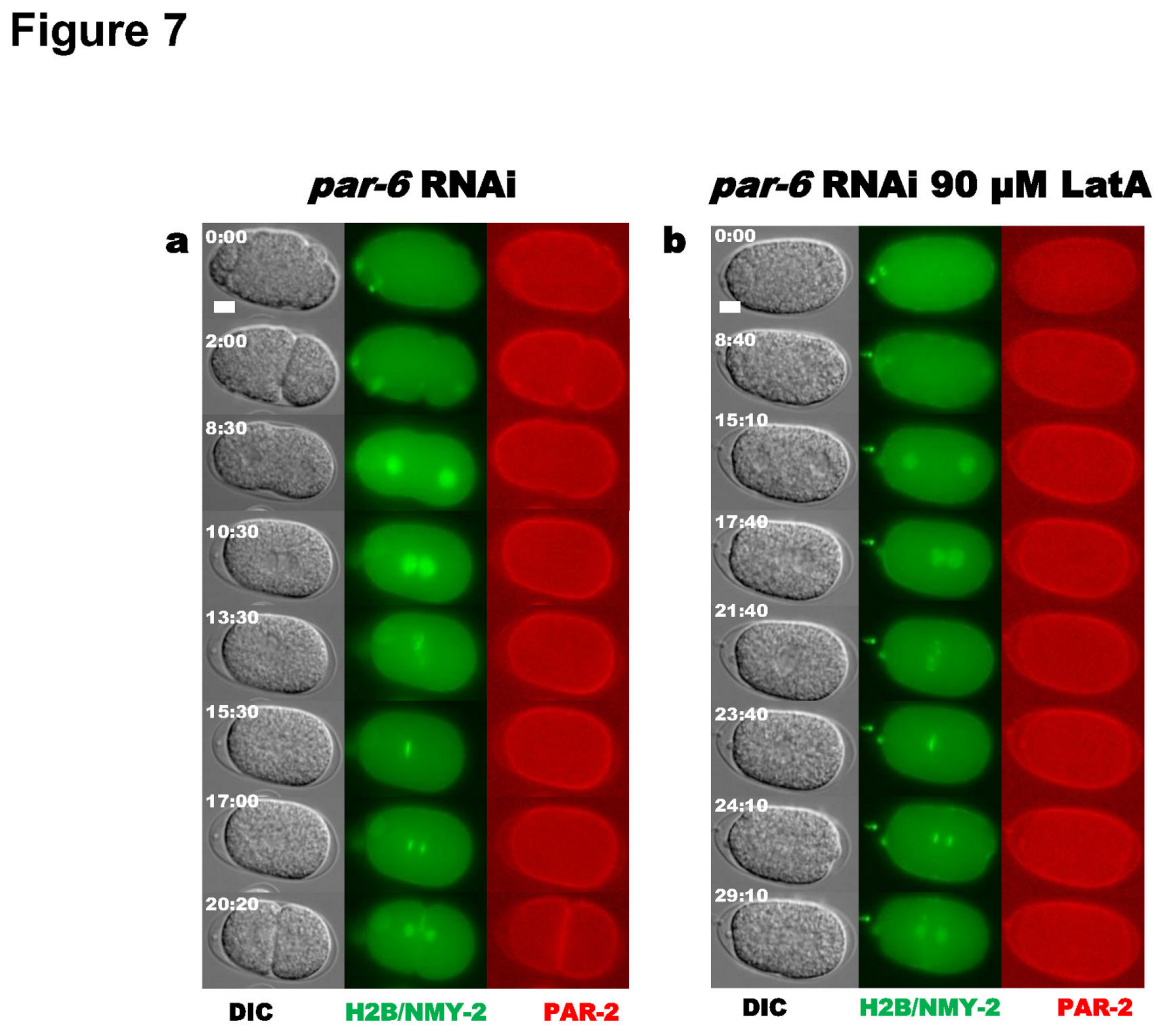
Effect of Latrunculin A on PAR-2 localization in PAR-6-depleted embryos. (**a**) par *6* RNAi treatment for 24 hr (*N* = 5). (**b**) par *6* RNAi treatment for 24 hr combined with 90 µM LatA injection following meiosis II (*N* = 4). For each panel, columns from left to right: DIC, NMY-2::GFP/ GFP::H2B and PAR-2::mCherry.

In order to determine whether microfilaments might play a direct role in cortical PAR-2 localization, we combined par *6* RNAi depletion with 90 µM LatA injections. In single-cell embryos depleted of PAR-6, without LatA treatment, PAR-2 localized uniformly throughout the cortex (*N* = 5) (Figure 7a) consistent with the known role for anterior PAR proteins in excluding PAR-2 from the cortex [7,8,9,44,45]. Injection of 90 µM LatA, following the completion of meiosis II, into par *6* RNAi embryos, had no additional effect on cortical PAR-2 localization; PAR-2 localized to the entire cortex (4 out of 4 embryos; Figure 7b). Thus LatA does not prevent PAR-2 from associating with the cortex, but rather interferes with the proper clearing of PAR-6 from the posterior cortex. Our experiments suggest that in LatA-treated embryos, the uniform distribution of the anterior PARs prevents PAR-2 from localizing to the cortex. In embryos with PAR-6 but lacking microfilaments, PAR-2 then localizes to the centrosomes by default perhaps due to its ability to bind microtubules [15].

Because our treatments with LatA resulted in failure to establish polarity, we also treated embryos expressing GFP-tagged beta-tubulin (GFP::TBB-2) with LatA to determine whether our LatA injections might compromise the microtubule system in addition to microfilaments, but we observed no detectable difference in microtubule morphology (7 out of 7 embryos; Figure S1). The orientation of anaphase showed no deviation from control embryos indicating that microtubules were still capable of stably anchoring to the cortex. This indicates that when the microfilament system is severely compromised by LatA, microtubules remain intact but are not sufficient to initiate cortical PAR-2 localization.

## Discussion

The use of carbon-reinforced nanopipettes to penetrate the durable eggshells of single-celled *C. elegans* embryos has provided a new technique with which to examine a very specific developmental event: the earliest stage of polarity establishment. By injecting well-defined doses of drugs that target the microtubule or microfilament cytoskeleton, we found that CRNPs are exceptional tools to investigate the development of *C. elegans* embryos in a temporally controlled manner and that the procedure itself does not perturb early embryonic development.

There are a number of advantages of CRNPs over existing techniques for circumventing the robust permeability barrier to expose *C. elegans* embryos to small molecules. A common existing technique is pressure-permeabilization, or ‘popping’, of the eggshell. In order for embryos to survive this technique the eggshell must be fully formed prior to the application of pressure to the embryo thus limiting how early embryos can be experimentally manipulated. CRNP injection extends the window of time that is experimentally accessible. Each injection takes approximately 30 seconds from initial piercing of the embryo to the retrieval of the pipette, allowing for rapid experimental manipulation (Video S1). A second, similar, method for embryo permeabilization is laser perforation of the eggshell [46]. Both methods rely on the diffusion of small molecules in the surrounding buffer into the embryo. The advantage of injection over this method is the ability to easily titrate the drug dosage. This can be difficult to do with the current diffusion-dependent methods as it is unclear whether the embryos equilibrate with the surrounding media or if a more complex drug diffusion process occurs. As we have shown (Figure 5), CRNPs allow titration of the molecule concentration for the assessment of a range of phenotypes. A third method for permeabilizing the eggshell is to utilize RNAi to generate “leaky eggshells” [25,26]. Some proteins that, when depleted, result in permeabilized embryos have also been implicated in embryo polarization and thus their knockdown may complicate studies of polarity establishment [37,47,48], reviewed by Johnston and Dennis [49]. The use of RNAi to create a permeable eggshell may also interfere with RNAi knockdown of additional targets, as combined RNAi can dilute depletion efficiency [50]. In principle, our CRNP technique could be used to introduce any number of drugs in combination with RNAi (Figure 6). Direct injection with micropipettes also allows delivery of a wider range of molecules than does iontophoresis, which requires a current to move small, charged molecules into the cell [51]. CRNPs are much stronger than conventional glass micropipettes and their hydrophilic lining facilitates transfer of aqueous solutions into cells by pressure-driven injection.

Polarity establishment in *C. elegans* provided a precise time point in which to establish CRNPs as a viable tool for cell biology. The fine temporal control of injection by CRNPs, coupled with live cell imaging, permitted us to take a closer look at the very early developmental time in which polarity is established. Our experiments reinforce a required role for microfilaments in polarity establishment and suggest that astral microtubules are not sufficient to establish polarity in the absence of actomyosin-mediated cortical reorganization.

When microfilaments were disrupted immediately following meiosis II by injection of a high dosage of LatA (90 µM), PAR-2 was unable to accumulate at the cortex and instead localized to the centrosomes (Figure 5), confirming that microfilaments are necessary for the initial establishment of the cortical PAR-2 domain [38]. Our examination of PAR-6 localization following LatA injection indicates that exclusion of PAR-2 from the cortex is most likely due to a failure to clear PAR-6 from the posterior cortex (Figures 6 and 7). The ability of PAR-2 to stably localize to the cortex in embryos depleted of PAR-6 by RNAi even after LatA treatment lends further support to mutual exclusion between anterior and posterior polarity proteins in models of polarity establishment. Additionally, these experiments demonstrate that microfilament depolymerization disturbs neither the ability of PAR proteins to bind the cortex, nor the ability of the anterior proteins to exclude PAR-2 from the cortex (Figures 6 and 7). Consistent with our findings, uniform retention of PAR-6 or PAR-3 at the cortex has been observed when NMY-2 is compromised by RNAi depletion [8,52]. Our LatA experiments have shown that disruption of the microfilaments’ ability to initiate PAR-6 clearing results in complete failure of PAR-2 to access the posterior cortex. This is consistent with models for polarity in which actomyosin flows transport PAR-3/PAR-6/PKC-3 to the anterior of the zygote to initiate their asymmetric localization, and the resulting nascent PAR-2 domain maintains that asymmetry [2]. However, the stronger effect of LatA on polarity establishment than that produced by *mlc-4* RNAi treatment [15,38,43] may suggest that actin microfilaments or actin-associated proteins direct polarity establishment by a means other than simply regulating actomyosin contractility.

The results of our LatA treatments do not support a model in which microtubules direct the self-organization of PAR asymmetry independently of actin dynamics in the *C. elegans* embryo. Other studies, using *mlc-4* RNAi treatment, have suggested that in the absence of actomyosin contractility, microtubules can facilitate cortical loading of PAR-2 by protecting PAR-2 from phosphorylation by PKC-3 [15,43]. However, we have found that when the actin cytoskeleton is severely disrupted by 90 µM LatA treatment, even fully intact microtubules are not sufficient for stable accumulation of PAR-2 at the cortex (Figure 4). Although it is possible that at this concentration we are seeing off-target effects of the drug, these results suggest that microtubule-directed polarity establishment in *C. elegans* requires intact microfilaments. This raises two possibilities: 1) that residual low levels of actomyosin contractility after *mlc-4* RNAi are sufficient to facilitate microtubule-dependent loading of PAR-2 or 2) that a role for actin filaments that is independent of myosin contractility is required for PAR-2 loading.

We anticipate that CRNP injection may find broad applications in future studies of *C. elegans* early embryonic development because of its ability to precisely target a specific developmental stage. Although our current application focuses on the single-cell C*. elegans* embryo, the sharp and strong CRNPs are capable of precise penetration through the eggshells of nematode embryos at any developmental stage and could also be applied for injection into other types of cells with tough coverings. With minimal modification to CRNPs, intracellular spatial control could also be achieved by using functionalized nanoparticles or liposomes to deliver drugs of interest [53]. Our injection method using CRNPs, which minimizes cellular damage while allowing precise manipulation, should be adaptable for a wide range of further applications in cell biology.

## Methods

### Pipette fabrication

Figure 1a shows the protocol steps for making CRNPs, adapted from previous studies [32,54]. Quartz capillaries (Sutter Instrument; Q100-70-7.5) were filled, via capillary action, with ~115 µL of catalyst solution (18 mg Fe(NO_3_)_3_ in 25 mL isopropyl alcohol) and laid flat to air dry at 21°C for at least 12 hours. They were then pulled with a pipette puller (Sutter Instruments P2000, parameters are (Heat, Filament, Velocity, Delay, Pull) = (700, 4, 55, 130, 55)_line1_ and (H, F, V, D, P) = (700, 4, 55, 130, 250)_line2_) into nanopipettes with outer and inner tip diameters of 135 ±66 nm and 74 ± 939 nm, respectively (*N* =80). A quartz tube ~1 inch in diameter and ~2 inches long was used to hold the pulled pipettes during carbon deposition. The pulled pipettes were placed in a chemical vapor deposition (CVD) furnace (Kevek Innovations, USA) to deposit a layer of amorphous carbon on the interior surface of the pipettes (6 h; 0.9 Standard Liter per Minute (SLM) CH_4_ and 0.6 SLM Ar at 920°C). Figure 1b shows an image after CVD, acquired using a LEO 1550 Field Emission Scanning Electron Microscope (Zeiss, Oberkochen, Germany). CRNPs were fabricated in batches of ~80, stored at room temperature (21°C) and ambient pressure, and front-loaded with solutions of interest before each injection experiment (see below).

To prepare holding pipettes, unmodified quartz capillaries, were pulled to a ~15 µm tip with parameters (H, F, V, D, P) = (700, 5, 100, 250, 100). These pipettes were used without subsequent modification as holders to maintain embryo position during piercing and injection.

### Preparation of Small Molecules and CRNP Loading

DAPI (D8417, Sigma) was dissolved in water to a final concentration of 2 mg/mL and stored at 4°C. Latrunculin A (L5163, Sigma) was dissolved in DMSO to a final concentration of 2.4 mM, aliquoted and stored for a maximum of 3 months at -20°C. All small molecule stock solutions were further diluted in 0.8X Egg Buffer (EB) + 0.5mM polyvinylpyrrolidone (PVP; P2307, Sigma) [19] (1X EB: 118 mM NaCl, 48 mM KCl, 2 mM CaCl_2_, 2 mM MgCl_2_) to a final concentration of 0.2 mg/mL for DAPI and 60 µM or 90 µM for LatA. YOYO-1 1mM stock solution (Y3601, Invitrogen) was diluted in 1X egg buffer to a final concentration of 1µM. Working solutions were kept at 4°C for no more than 2 days.

CRNPs were frontloaded by applying suction with a hand-held syringe, for 3 minutes. Loaded CRNPs were then mounted on a manipulator and dipped into a droplet of injection solution and left to equilibrate for 3 minutes. CRNPs were attached to a Femtojet unit (Eppendorf, Hamburg, Germany) to control duration and pressure of injection. A second micromanipulator, opposite the mounted CRNP, was use to manipulate the holding pipette. This holding pipette was attached via plastic tubing to a 50 ml syringe, which could be used to apply suction to hold the embryo in place during CRNP penetration and removal.

### *C. elegans* strains and maintenance

*C. elegans* strains were maintained by standard techniques [55], at room temperature (21-23°C) before isolation of the embryos. N2 (Bristol) was used as the wild-type strain. Strains expressing fluorescently tagged proteins of interest have the following genotypes:

TY3558 *unc-119*(*ed 3*) *ruIs32*[*Ppie-1::GFP::his-11 + unc-119(+*)] *III; ojIs1*[*pie-1::GFP::tbb-2 + unc-119(+*)]

KK1177 *unc-119*(*ed 3*) *ruIs32*[*Ppie-1::GFP::his-11 + unc-119(+*)] *III; axIs1929* [*pFM033 pie-1::NMY-2::GFP and mCherry::PAR-2, unc-119(+*)]

KK1169 *itIs272*[*Ppar-6::PAR-6::mCherry + unc-119(+*)]*; itIs153[pie-1::PAR-2::GFP* (*pMW1.03, pRF4 rol-6*] (provided by Alex Beatty, Cornell University).

*itIs153* is from Cuenca et al. (2003) [8]; *itIs272* is an mCherry derivative of pJN284 [56] provided by Heon Kim, Cornell University, and *axIs1929* is from Zonies et al. (2010) [43]. TY3558 was provided by the Caenorhabditis Genetic Center, which is funded by the National Institutes of Health Office of Research Infrastructure Programs (P40 OD010440).

### RNAi treatment

RNAi was performed by feeding [57]. HT115(DE3) bacteria were freshly transformed with RNAi plasmids, grown to log phase, induced with 400 µM IPTG for 2.5 hours, concentrated five-fold and spotted onto agar plates containing minimal medium with 12.5 µg/mL tetracycline and 50 µg/mL carbenicillin. Worms were allowed to feed on RNAi bacteria at least 16 hours before embryo isolation. L4440 empty vector was used as a negative control [58]. The par *6* RNAi construct is from Aono et al.(2004) [59].

### Injection experiments

One-cell embryos were isolated from young adult worms by dissection in 0.8X EB + 0.5 mM PVP on a glass coverslip. A Ti-E Nikon inverted microscope (Nikon Instruments, Melville NY USA) was used for imaging. A holding pipette was used to immobilize the embryo and the embryo was oriented such that the CRNP would pierce the anterior end as determined by polar body position. Embryos were injected immediately following meiosis II, as assessed by GFP::histone visualization. Duration of injection was set to 1 second, and injection pressure ranged from 10 to 20 psi. Injection success was verified by assessing the morphological phenotypes produced by LatA or by dye visualization. Each CRNP was used to inject multiple embryos.

Application of suction to the holding pipette allowed CRNP withdrawal after injection. Nomarski and fluorescence (FITC, C-FL Texas Red HYQ or DAPI, Nikon filters) images were acquired (NIS Elements, Nikon) sequentially every 30 sec until either cytokinesis or the equivalent cell cycle stage was reached or 1 h elapsed, whichever occurred first.

Embryos injected with YOYO-1 were transferred using a large quartz pipette to a fresh drop of 0.8X + 0.5 mM PVP on a glass coverslip for further development. The drop was kept in a humid chamber for up to 24 hours or until hatching.

For the injection volume calibration, we injected 1.25 mg/ml of dextran-TxRED (~3000 MW) (Invitrogen) into glycerol. The fluorescence image of dextran immediately following injection completion (1 s after injection was initiated) was used to calibrate the injection volume. An intensity line scan of the injected drop of the image was fit to a Gaussian function and the standard deviation of the Gaussian was used to estimate the radius of the spherical drop.

## Supporting Information

Figure S1 **Effect of Latrunculin A treatment on microtubule formation.** (**a**) Control with injection buffer containing 3.75% DMSO (*N* = 8). (**b**) Injection of 60 µM LatA (*N* = 7). In each panel, DIC is on the left and GFP::H2B/ GFP::TBB-2 is on the right. Below each panel is an enlargement showing the maximal size of GFP::TBB-2 at the centrosomal region, indicated by the white arrowheads.
(TIFF)

Video S1 **A real-time video showing CRNP piercing of a single-cell embryo.** The CRNP pierced the future anterior end (left) of a *C. elegans* one-cell embryo. Suction applied by a quartz holding pipette (right) stabilized the embryo, allowing retrieval of the CRNP. Images were taken at 2 frames/second under DIC. Scale bar represents 20 µM.
(AVI)
