# Supplementary figures and images for: Small Molecule Injection into Single-Cell C. elegans Embryos via Carbon-Reinforced Nanopipettes
Source: PLoS One. 2013 Sep 26;8(9):e75712. doi: 10.1371/journal.pone.0075712 (PMC3784451; doi:10.1371/journal.pone.0075712)

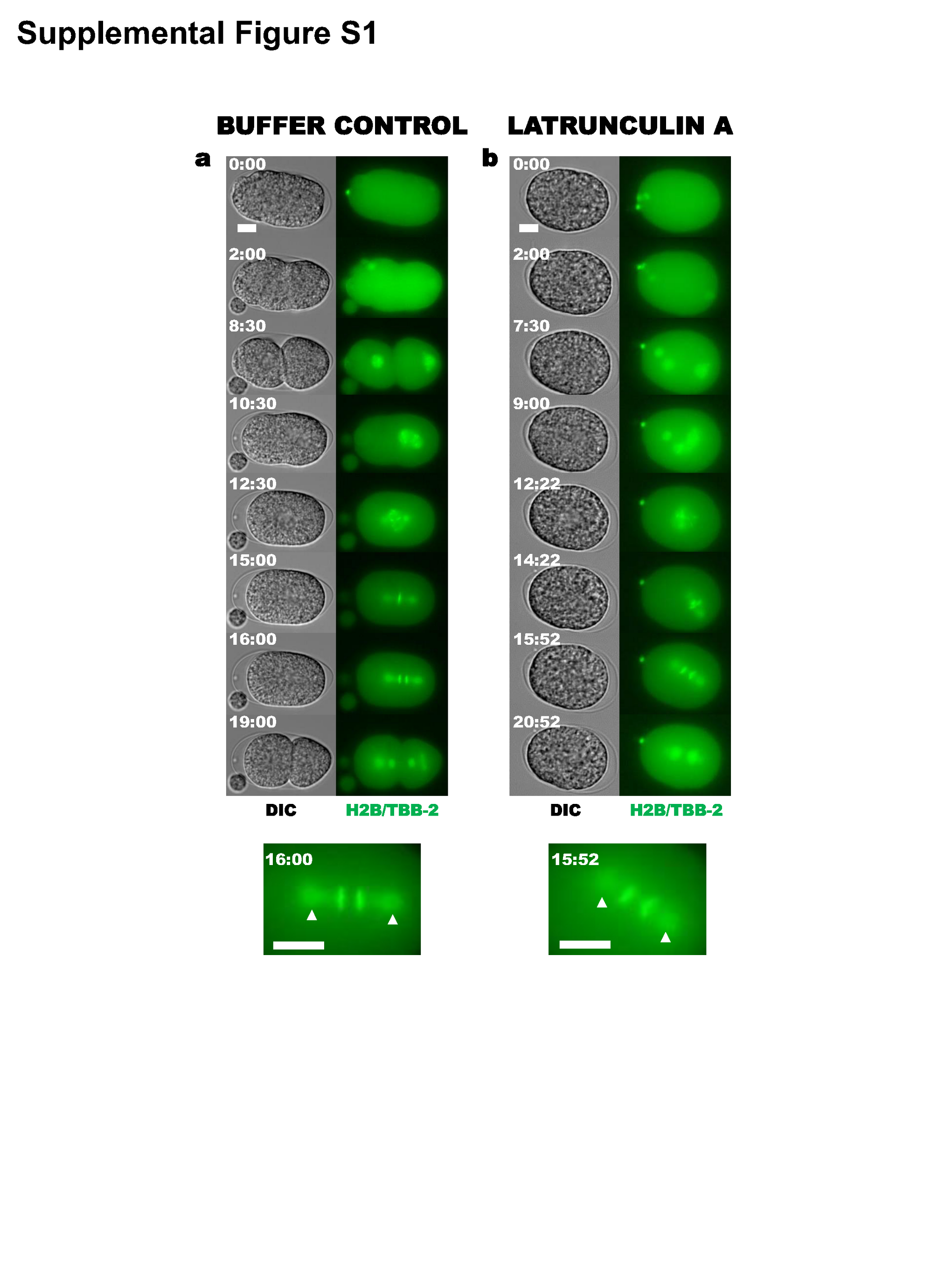

Supplement: Figure S1 — Effect of Latrunculin A treatment on microtubule formation. (a) Control with injection buffer containing 3.75% DMSO (N = 8). (b) Injection of 60 µM LatA (N = 7). In each panel, DIC is on the left and GFP::H2B/ GFP::TBB-2 is on the right. Below each panel is an enlargement showing the maximal size of GFP::TBB-2 at the centrosomal region, indicated by the white arrowheads. (TIFF) [file pone.0075712.s001.tiff]
